# Intra-capsular Fracture of the Neck of the Femur

**Published:** 1877-12

**Authors:** H. Wardner

**Affiliations:** Cairo, Ills.


### INTRA-CAPSULAR FRACTURE OF THE NECK OF THE FEMUR. RECOVERY.

By H. Wardner, M. D., of Cairo, Ills.

[A case reported to the Southern Illinois Medical Association at Anna, June, 1877.]

Medical literature seldom furnishes a record of intra-capsular fracture of the femoral neck occurring in a young person. It is even asserted by most surgical authors that it does not occur. Yet a few cases have been reported, showing that there are exceptions to this as to most general rules.

There may be interstitial changes resulting from disease, or injury, or both combined, by which the power of resistance in the bony structure is so diminished, as to render the person liable to the accident from apparently trifling causes.

The following case of a patient, to whom I was called on the 1st of December, 1876, was undoubtedly one of this class.

The subject of the accident was a lad between fourteen and fifteen years of age, of rather delicate constitution. For a year or more he had suffered from a "fever sore" on the left leg, involving to some little extent the integrity of the tibia, but from which he was recovering at the time.

On the 5th of November, 1876, he was playing with two other lads who had hold of one end of a rope, he having hold of the other and being in the rear, when they all ran down a declivity two or three feet in extent. When the patient came to the edge, the boys in front suddenly jerked the rope, so that he partly jumped and partly fell down the declivity, striking however, upon his feet.

He at once perceived that he had injured the right hip, yet was able to walk to his home, and subsequently to move about, doing light work, upon one occasion even holding the plow. Although he walked with some difficulty, and in an un natural manner, he continued to exert himself to this extent for twenty-four days.

On the morning of the 29th of the same month, upon attempting to get out of bed, one foot became entangled in the bed-clothing, and this led him to exert forcibly the adductor muscles, when he suddenly cried out with pain, saying his "hip had gone out of place," and he found himself unable to rise.

Dr. H. S. Smith, of Blandville, Ky., was at once summoned. Upon the supposition that a dislocation had been produced, he made efforts at reduction, but without success.

On the 30th Dr. D. P. Juett was called in consultation, and he diagnosticated dislocation of the head of the femur upon the dorsum ilii. The patient was anæsthetized with chloroform, and repeated attempts were made at reduction by manipulation, using the femur as a lever, but so unsatisfactory was the result, that it was decided to secure further advice.

In answer to a note from Dr. Juett, I visited the patient on the evening of the 1st of December, with a view to consultation with that gentleman and Dr. Smith. I found the lad lying upon his back suffering severe pain. Considerable inflammation had been excited by the attempts at reduction.

The leg was shortened from one to two inches, lying nearly paralell with the opposite limb, the toes everted.

The trochanter of the affected limb was between one and two inches higher than that on the sound side, and turned backward so as to lose the prominence seen in the natural position. It rotated with the limb, so as to describe the arc of a circle somewhat less than natural, but not to a marked degree. There was no effusion or apparent injury to the soft parts about the trochanter. In consequence of the previous attempts at reduction, the parts had become very sensitive. After chloroform was administered, I seized the limb and found it very mobile and easily extended to the full length, when its aspect became entirely normal. A dull crepitation was distinctly recognized in the joint.

The diagnosis of intra-capsular fracture of the femoral head was established, and the treatment adopted at the time, and subsequently continued, was as follows: The patient being placed upon his back on a smooth bed, the foot of which was elevated about four inches, a splint, made after the manner of Hodgen's extension apparatus, was applied to the limb which was elevated ten to fifteen degrees, or until the extending force became sufficient to overcome the shortening, so that when a straight rod four feet long was applied against both heels, it was perpendicular to the axis of the body. The hip now assumed a natural appearance, the trochanters corresponding in position and prominence, and the fragments were thus believed to be in apposition. A wide flannel bandage was then snugly applied about the hips. The lad now said he was free from pain and soon fell asleep. The extension was kept up continuously for fifty-five days. On the fortieth day, a plaster of Paris dressing was applied about the hip, so as to prevent motion, the extending apparatus being removed fifteen days later. The plaster dressing was removed on the seventy-second day, and he was furnished with a pair of crutches. His health remained good during the entire time of the confinement. Dr. Smith writes me that he saw the patient on the 17th of March, 1877, when he was walking by the aid of his crutches. The injured limb was of the same length as the sound one, and no deformity of any kind was detected. On the 1st of May following, he was reported able to walk without crutch or stick. His friends, who were quite intelligent, appreciated the importance of the case, and expressed great satisfaction at the result.

The interesting features of this case are: the age of the patient; the time from the original injury to the date of the complete fracture; the mode of treatment; and the result.

As to the age in which this fracture occurs, Dr. Gross says: "Experience has shown that it (the neck of the femur) is subject to fracture, as a general rule, only after the age of fifty, when its spongy and compact tissues suffer from atrophy and fatty degeneration, thus rendering it more or less brittle and incapable of withstanding injury."

In a collection of 250 cases made by Sir Astley Cooper, the youngest patient was thirty-eight years old.

Erichsen refers to a case reported by Mr. Stanley, occurring in a young lady of eighteen years.

Dr. F. H. Hamilton mentions four cases of suspected fracture, or separation of the epiphysis, in persons aged respectively eleven, eighteen, sixteen, and fifteen; but he remarks that "at least one dissection is required to confirm the diagnosis in any of them."

Authors in surgery inform us that the fracture may be denticulated, or the fragments so dovetailed together, that they may not separate for some days; or we may have an impacted or partial fracture, especially in younger persons, in whom the animal constituents of the bone are abundant. The latter injury is more apt to occur when the force is received in or near the line of the axis.

This patient received the force of the fall or jump upon his feet, he did not fall upon his side. The neck of the bone at his age forms an obtuse angle with the shaft of about 135 degrees, so that the force was spent upon the neck rather in a longitudinal direction, than transversely. There was probably a partial fracture at first; but whatever the injury may have been, repair did not follow as soon as might be expected in a patient of his age; on the contrary, it was followed by pathological changes which rendered the bone brittle and easily broken, as it was.

In the treatment of this fracture, as of all others, two principal indications are to be kept in view; viz., apposition and fixation.

By suspending the limb in Hodgen's splint, the extension is principally made by its own weight, the counter extension being made by the weight of the body.

We can thus secure a constant and equable extension, and the patient can be allowed some liberty of motion and change of position, lessening very materially the danger from bed sores.

The bandage was first applied about the pelvis, without the plaster, so that frequent observation could be readily made of the parts, lest erysipelatous inflammation might follow the injury after the several attempts at reduction.

The plaster dressing well applied will keep the joint quite immovable.

By the extension, and plaster dressing, the fragments were kept in apposition until bony union was complete.

